# Impaired toll-like receptor 8 signaling in multiple sclerosis

**DOI:** 10.1186/1742-2094-10-74

**Published:** 2013-06-21

**Authors:** Tory P Johnson, Richa Tyagi, Karan Patel, Nicoline Schiess, Peter A Calabresi, Avindra Nath

**Affiliations:** 1 Section of Infections of the Nervous System, National Institute of Neurological Disorders and Stroke, National Institutes of Health, Bldg 10; 7C-103, 10 Center Drive, Bethesda, MD 20892, USA; 2 Department of Neurology, Johns Hopkins University, Baltimore, MD, USA

**Keywords:** Toll-like receptor, Multiple sclerosis, Gene expression, Interleukin 12, TLR8

## Abstract

**Background:**

The etiology and immunopathology of multiple sclerosis (MS) is not well understood. It is recognized that although autoreactive T cells are the main early mediators of disease, other cell types, including cells of the innate immune system contribute to MS pathogenesis. The objective of this study was to determine if Toll-like receptor (TLR) signaling is functionally altered in patients with MS.

**Findings:**

Peripheral blood mononuclear cells from healthy donors and patients with relapsing remitting MS were stimulated with specific agonists of TLRs 3, 7, 8 and 9. Using quantitative polymerase chain reaction transcript levels of tumor necrosis factor-α, interferon-α and interleukin (IL)-12β were quantified from patients with MS and healthy donors. TLR8-induced production of *IL12B* transcripts and protein was functionally impaired in patients with MS as compared to healthy controls (*P* <0.05 and *P* <0.005, respectively). Patients with MS also expressed lower baseline levels of TLR8 as compared to healthy controls (*P* <0.05).

**Conclusions:**

TLR8 expression and signaling is impaired in peripheral blood mononuclear cells from patients with MS. This finding suggests that loss of TLR8 signaling may be contributing to autoimmune processes in MS.

## Findings

Multiple sclerosis (MS) is an autoimmune disorder characterized by demyelination, chronic inflammation, and neuronal damage primarily caused by activated, auto-reactive CD4+ and CD8+ T cells directed against myelin [1]. Combinations of genetic and environmental factors, including pathogens, are believed to trigger the abnormal immune activation in MS [2]. Pathogens which initiate innate immune responses in the central nervous system (CNS) via Toll-like receptor (TLR) signaling may lead to the development of autoreactive T cells due to antigen spread in a process known as bystander activation [2,3].

TLRs are pattern recognition receptors (PRRs) that recognize conserved pathogen-associated molecular patterns (PAMPs) [4]. TLRs responsible for detecting viral PAMPs include TLR3, which detects viral double stranded (ds)RNA, TLR7 and TLR8, which detect viral single stranded (ss)RNA, and TLR9, which detects dsDNA and are located in the endosome [5,6]. TLR signaling results in the activation of T cells [7] and production of pro-inflammatory cytokines, such as interleukin (IL)-12β, interferon (IFN) and tumor necrosis factor (TNF)-α, which are known to be increased in patients with MS [5,8,9]. Many studies have implicated TLRs in the pathophysiology of MS and experimental autoimmune encephalomyelitis (EAE) [4]. For instance, activation of TLRs reliant on the downstream adaptor protein MyD88 (TLR7, 8 and 9) may enhance MS progression, while the activation of TLR3, which is not dependent on MyD88, may be protective in neurons [4,10]. However, it remains unclear if TLR signaling is functionally dysregulated in patients with MS prior to treatment. To better understand the role of TLR signaling in MS, we sought to determine the functional properties of TLRs 3, 7, 8 and 9 in these patients as compared to healthy donors.

**Figure 1:**
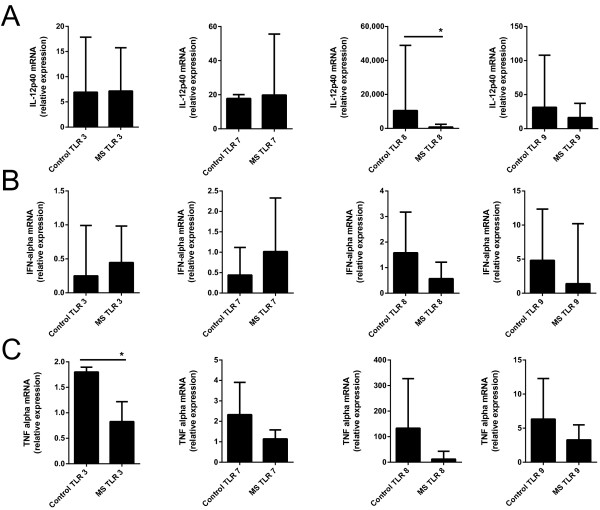
**PBMC cytokine gene expression levels after TLR stimulation.** (**A**) IL-12B transcripts were decreased in patients with MS as compared to healthy donors after Toll-like receptor (TLR) 8 stimulation (Student’s unpaired two-tailed *t*-test, * = *P* <0.05, n = 10 per group) but not after TLR 3, TLR 7 or TLR 9 stimulation. (**B**) There was no difference between patients with MS and healthy donors in IFN-alpha transcript production after TLR stimulation. (**C**) TNF-alpha transcripts were decreased in patients with MS as compared to healthy donors after TLR 3 stimulation (Student’s unpaired two-tailed *t*-test, * = *P* <0.05, n = 10 per group) but not after TLR 7, TLR 8 or TLR 9 stimulation. Data shown are median ± 25% to 75% interquartile range relative gene expression of each cytokine normalized to GAPDH and a reference sample. PBMC, peripheral blood mononuclear cells.

To determine if patients with MS had dysregulated TLR signaling we analyzed the transcriptional response of IL12β, IFN-α and TNF-α to TLR3, 7, 8 and 9 stimulation. Briefly, peripheral blood mononuclear cells (PBMC) were obtained from clinically stable untreated patients with relapsing remitting MS (n = 10) and from healthy age- and gender-matched donors (n = 10). Patients with MS were aged from 49 to 74 years (median 57 years; seven females and three males). Healthy donors ranged in age from 41 to 72 years (median 57.5; seven females and three males). All patients and donors gave informed consent and this study was approved by the Office of Human Subjects Protection and Research at the National Institutes of Health. PBMCs from healthy donors and patients with MS were isolated by a standard operating procedure using Ficoll-Isopaque density gradient centrifugation (Gibco, Life Technologies Ltd., Paisley, UK) and were immediately stored in liquid nitrogen until further use. Cells from controls and patients with MS were processed under the same protocol. Cells were maintained in Iscove’s Modified Dulbecco’s Media (Invitrogen, Carlsbad, CA, USA) supplemented with 10% (v/v) human serum (Sigma, St. Louis, MO, USA) and 1% (v/v) antibiotic-antimycotic mixture (Invitrogen) at 37°C with 5% CO_2_. PBMCs were plated at 1.5 × 10^6^ cells/well and treated with either media (unstimulated), phytohaemagglutinin (PHA) (0.025 μg/mL), TLR3 ligand, Poly(I:C) (10 μg/mL), TLR7 ligand, Imiquimod (25 μg/mL), TLR8 ligand, ssPolyU/LyoVec (10 μg/mL) or TLR9 ligand, ODN2216 (0.025 μg/mL) for six hours at 37°C. All ligands were obtained from InvivoGen (San Diego, CA, USA). After incubation, cells were collected and total RNA was extracted using the QIAGEN RNeasy Plus kit (Qiagen, Germantown, MD, USA). Up to 100 ng of RNA from each sample was reverse transcribed into cDNA per the manufacturer’s instruction using the SuperScript III First-Strand Synthesis SuperMix for quantitative-PCR (Invitrogen). Gene expression levels for *IFNA2*, *TNFA*, *IL12B* and glyceraldehyde 3-phosphotate dehydrogenase (*GAPDH*) were determined by quantitative PCR performed on a ViiA™ 7 Real-Time PCR System (Applied Biosystem, Carlsbad, CA, USA). All primers and standards were obtained from Origene (Origene, Rockville, MD, USA). Cytokine transcript expression levels were determined by relative quantification (ΔΔ Ct method [11]) for each individual using GAPDH for normalization. Cells from patients with MS showed a 58-fold decrease in transcript levels of *IL12B* in response to TLR8 stimulation (median ± 25% to 75% interquartile range = 179.3 ±± 56.0 to 896.0 versus 10,518 ± 1,607 to 48,932 relative expression; *P* <0.05) (Figure 1A), but no difference was detected after TLR3, TLR7 or TLR9 stimulation. No significant difference was found between patients with MS and healthy donors in production of *IFNA* with any TLR stimulation (Figure 1B). Patients with MS showed a two-fold decrease in transcript levels of *TNFA* in response to TLR3 stimulation (median ± 25% to 75% interquartile range = 0.9 ± 0.4 to 1.0 versus 1.8 ± 1.1 to 1.9 relative expression; *P* <0.05) as compared to healthy donors (Figure 1C), but not after TLR7, 8 or 9 stimulation.

**Figure 2:**
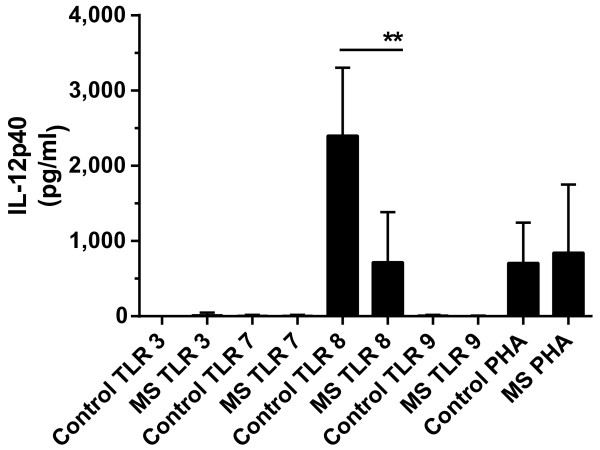
**IL-12p40 protein expression in PBMC.** IL-12p40 protein expression is decreased in patients with MS as compared to healthy donors after TLR8 stimulation (ANOVA with Bonferroni’s correction for multiple comparisons, ** = *P* <0.005, n = 10 per group), but IL-12p40 protein expression was not different between patients with MS and healthy donors after stimulation with PHA (n = 10 per group). No appreciable production of IL-12p40 occurred after stimulation with TLR3, 7 or 9. Data shown are the mean ± SEM IL-12p40 protein concentration in pg/ml. MS, multiple sclerosis; PBMC, peripheral blood mononuclear cells; PHA, phytohaemagglutinin; TLR, Toll-like receptor.

As MS is a hyper-inflammatory disorder and as IL-12 is known to be elevated in patients with MS [12], we were surprised to see a decrease in *IL12B* production from patients with MS. We, therefore, confirmed these results by determining the concentration of IL-12p40 produced after TLR8 stimulation. Briefly, 1.5 × 10^6^ cells were plated and TLR8 was stimulated as above for 24 hours. After incubation, supernatants were collected and analyzed for IL12p40 protein secretion by enzyme-linked immunosorbent assay (ELISA) (Cell Sciences, Canton, MA, USA). In accordance with the transcription data, we found a decrease in IL12p40 protein secreted from PBMCs from patients with MS as compared to healthy controls after TLR8 stimulation (mean ± SE = 717.7 ± 211.3 versus 2,401 ± 285.4 pg/ ml; *P* <0.005) but not after PHA stimulation (mean ± SE = 843.5 ± 287.6 versus 709.4 ± 169.8 pg/ ml; *P* >0.05) (Figure 2) indicating the cells from patients with MS were capable of responding to the same level as cells from healthy donors. No appreciable production of IL12p40 was observed after stimulation with TLR3, 7 or 9 (Figure 2).

**Figure 3:**
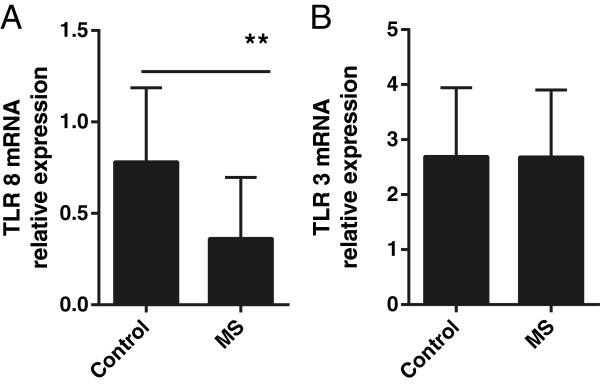
**TLR 8 and 3 gene expression in PBMC.** Patients with MS and healthy donors differ in baseline (**A**) TLR8 transcript expression (Student’s unpaired two-tailed *t*-test, ** = *P* <0.005, n = 10 per group) but not in (**B**) TLR3 transcript expression (n = 7 per group). Data shown are median ± 25% to 75% interquartile range relative TLR mRNA quantification at baseline for each individual normalized to GAPDH and a reference sample. MS, multiple sclerosis; PBMC, peripheral blood mononuclear cells; TLR, Toll-like receptor.

We next analyzed the baseline expression of TLR8 and TLR3 in patients with MS as well as healthy donors. Using RNA harvested from unstimulated cells as described above, quantitative PCR was performed using primers specific for TLR8 or TLR3 and GAPDH. TLR transcript expression levels were determined by relative quantification as described above. Cells from patients with MS showed a 2.7-fold decrease in transcript levels of TLR8 (median ± 25% to 75% interquartile range = 0.3 ± 0.1 to 0.6 versus 0.8 ± 0.6 to 1.2 relative expression; *P* <0.005) (Figure 3A); no difference in TLR3 expression was detected (median ± 25% to 75% interquartile range = 2.8 ± 1.5 to 3.7 versus 2.7 ± 1.7 to 4.0 relative expression; *P* >0.05) as compared to healthy donors (Figure 3B).

## Conclusions

This study shows that patients with MS exhibit a profound dysregulation of TLR8 signaling as compared to healthy donors manifested by IL-12β transcript and IL-12p40 protein production. The mechanism of depressed responsiveness of TLR8 appears to be a result of decreased TLR8 baseline expression in PBMC from patients with MS. Our data indicate that TLR8 signaling in patients with MS may be functionally hypoactive, resulting in detectable under-production of IL-12β. *IL12B* encodes the p40 subunit of IL-12 which forms both IL-12 and IL-23, two cytokines that are considered a driving force in EAE progression [13-16]. Although IL12p40 appears to have a critical role in EAE, MS pathogenesis may not be dependent on IL-12p40. A clinical trial using ustekinumab, a monoclonal antibody against IL-12p40, in patients with MS showed no beneficial clinical effect [16]. Therefore, the lack of IL12p40 detected in our study does not conflict with current data, although it seems counter-intuitive in an auto-immune mediated disease. Our study also detected a statistical difference in TNFα production after TLR3 stimulation; however, the expression values were low and the difference was rather modest, hence its biological relevance is unknown. We looked specifically at functionality of a subset of TLRs with a limited cytokine output. Our study is unique because we studied untreated patients in the absence of a clinical relapse. We readily detected a robust difference in TLR8 function between patients with MS and healthy donors. This finding may be clinically relevant as TLR8 deficient mice have increased autoimmunity [17]. Interplay between TLR8 loss and hyper-expression of TLR7 has been documented in mice [17] and patients with MS who are treated with INF-β1 therapy have an increase in TLR7 expression [18]. TLR8 is the least well understood of the human TLRs and our study provides additional evidence that TLR8 needs further investigation and, similar to the murine model, that human TLR8 deficiency may contribute to autoimmune processes.

## Abbreviations

CNS: Central nervous system; Ds: Double stranded; EAE: Experimental autoimmune encephalomyelitis; ELISA: Enzyme-linked immunosorbent assay; GAPDH: Glyceraldehyde 3-phosphotate dehydrogenase; IFN: Interferon; IL: Interleukin; MS: Multiple sclerosis; PAMP: Pathogen-associated molecular patterns; PBMC: Peripheral blood mononuclear cells; PHA: Phytohaemagglutinin; PRR: Pattern recognition receptors; Ss: Single stranded; TLR: Toll-like receptor; TNF: Tumor necrosis factor.

## Competing interests

All authors declare that they have no competing interests.

## Authors’ contributions

TPJ designed, performed and analyzed the experiments, coordinated collaborations and wrote the manuscript. RT and KP performed and analyzed experiments. NS designed the experiments and conducted the pilot studies. PAC assisted in experimental design. AN conceived of the project, designed and analyzed the experiments, coordinated collaborations and wrote the manuscript. All authors read and approved the final manuscript.
